# Central nervous system-specific efficacy of CDK4/6 inhibitors in randomized controlled trials for metastatic breast cancer

**DOI:** 10.18632/oncotarget.27238

**Published:** 2019-10-29

**Authors:** Long V. Nguyen, Karlee Searle, Katarzyna J. Jerzak

**Affiliations:** ^1^ Division of Medical Oncology and Hematology, Sunnybrook Odette Cancer Centre, University of Toronto, Toronto, ON, Canada

**Keywords:** breast neoplasms, blood-brain barrier, palbociclib, ribociclib, abemaciclib

## Abstract

**Importance:** Metastatic breast cancer with central nervous system (CNS) metastases carries a poor prognosis. Recently, CDK4/6 inhibitors have demonstrated a progression free survival (PFS) and overall survival benefit when combined with standard endocrine therapy in advanced hormone receptor (HR)+/HER2- breast cancer. Pre-clinical data suggests possible activity of CDK4/6 inhibitors in the brain, but their CNS-specific benefit has not been explored in clinical practice.

**Methods:** We reviewed clinical trials investigating the efficacy of CDK4/6 inhibitors for advanced or metastatic HR+/HER2- breast cancer. We also reviewed pre-clinical studies that demonstrated the ability of CDK4/6 inhibitors to cross the blood-brain barrier (BBB) and halt the growth of brain metastases in animal models.

**Findings:** An ongoing phase II trial (NCT02308020) was designed to investigate the safety and tolerability of abemaciclib for treatment of patients with CNS metastases, with preliminary data showing partial response in some patients. Review of key randomized phase III trials revealed a scarcity of data pertaining to the development of new CNS metastases. Pre-clinical models demonstrate that CDK4/6 inhibitors are able to cross the BBB and can delay the growth of brain metastases.

**Conclusions:** Despite encouraging pre-clinical evidence, there is a lack of clinical data to inform CNS-specific response rates to CDK4/6 inhibitors among patients with metastatic breast cancer. Given that the treatment of patients with breast cancer brain metastases represents an area of unmet medical need, enrollment of patients with CNS metastases in ongoing clinical trials should be encouraged; innovative trials that examine response of CNS metastases to CDK4/6 inhibitors are also of interest.

## INTRODUCTION

Breast cancer with central nervous system (CNS) metastases portends a very poor prognosis. Treatment options are often limited to radiation and/or surgery given the poor penetration of most systemic agents through the blood-brain barrier (BBB). Given a high morbidity and mortality associated with brain metastases, the identification of systemic therapies to optimize treatment of CNS disease and/or prevent the development of CNS metastases among patients with metastatic breast cancer is of interest.

**Figure 1:**
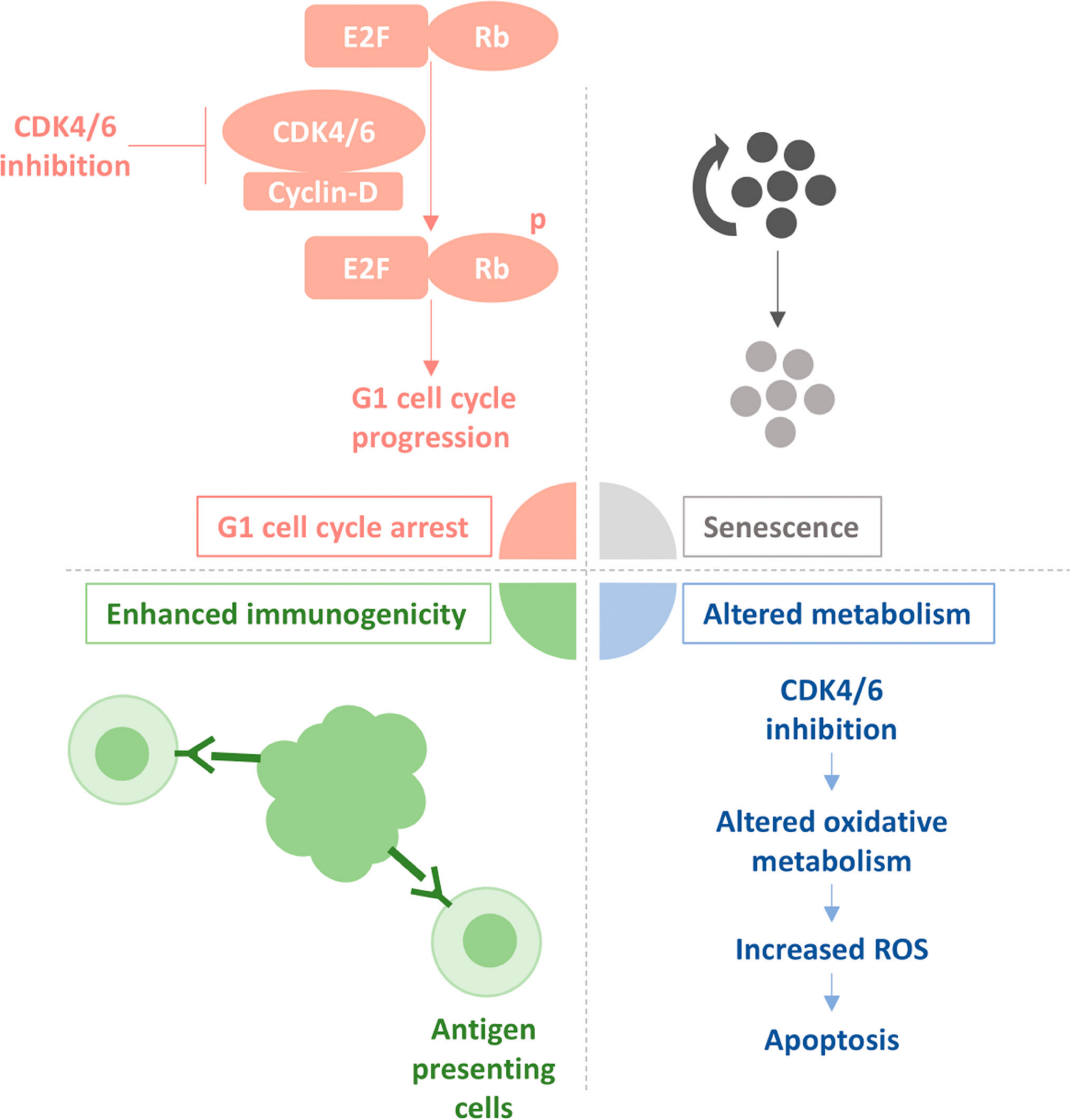
Effect of CDK4/6 inhibition on Rb-proficient cancer cells. The schematic presented depicts how CDK4/6 inhibitors prevent phosphorylation of the retinoblastoma (Rb) tumor suppressor protein by disrupting the association of Cyclin-D and CDK4/6. This causes unphosphorylated Rb to stay bound to E2F, a family of transcription factors that when decoupled from Rb leads to G1-to-S transition, and cell cycle progression. This process further leads to senescence and growth arrest of rapidly dividing tumour cells. Also depicted is how CDK4/6 inhibition causes cell death by enhancing the recognition of tumour antigens by antigen-presenting cells, as well as altering oxidative metabolism, resulting in an increase of reactive oxygen species (ROS).

CDK4/6 inhibitors are ATP-competitive inhibitors of cyclin-dependent kinases CDK4 and CDK6 that have shown efficacy for treatment of patients with hormone receptor (HR)+/HER2- advanced breast cancer [1]. CDK4 and CDK6 are aberrantly activated in several cancers, resulting in increased cellular proliferation due to loss of regulation of the G1 cell cycle checkpoint. Their inhibition triggers cell cycle arrest, increased senescence, and as a result, increased cell death [2] (Figure 1). The recent discovery that CDK4/6 inhibitors may have CNS activity in pre-clinical models is intriguing [3–6], raising the potential for an additional modality for the treatment of breast cancer brain metastases.

Here, we review the key clinical trials that investigated the combination of CDK4/6 inhibitors and standard endocrine therapy among patients with HR+/HER2- metastatic breast cancer, and report previously unpublished data from these trials regarding outcomes of patients with CNS metastases. Furthermore, we review pre-clinical evidence that suggests CNS-specific activity of CDK4/6 inhibitors.

## RESULTS

### Evidence from clinical trials

A recent phase II trial (NCT02308020) was specifically designed to study the effect of abemaciclib, a CDK4/6 inhibitor, in heavily pre-treated patients with CNS metastases [7]. This study included cohorts of patients with CNS metastases from HR+/HER2- and HR+/HER2+ breast cancer, non-small cell lung cancer, and melanoma, all of whom received treatment with abemaciclib. There was no control arm in this particular study. Patients with evidence of CNS progression but stable extra-cranial disease for 3 months or longer were enrolled, but those with leptomeningeal disease were excluded (Table 1). Preliminary results from a total 52 patients with HR+/HER2- CNS metastases are currently available. Twenty (38.5%) patients had a decrease in the sum of their intracranial target lesions [7]. However, based on the Response Assessment in Neuro-Oncology Brain Metastases (RANO-BM) criteria [8], only 3 patients (5.8%) demonstrated a partial response and 10 patients (19.2%) demonstrated stable disease persisting for ≥6 months; this translates into a clinical benefit rate of 25% (13 of 52 patients). The overall median progression free survival (PFS) was 4.4 months (range 2.6 – 5.5 months) [7].

**Table 1:** Summary of primary outcomes, and outcomes for patients with CNS metastases in clinical trials investigating the efficacy of CDK4/6 inhibitors

CDK4/6 inhibitor	Trial	Study type	Study population	Interventions	Overall outcomes	Brain-specific outcomes
Abemaciclib	NCT02308020 [7]	Phase II	- HR+/HER2-	Abemaciclib +/− ET	- Overall median PFS from both CNS and visceral disease = 4.4 months (range 2.6 – 5.5 months)	- 52 patients with known brain metastases
			- CNS metastases			- 3/52 patients (5.8%) showed confirmed intracranial response
			- Previous CT, RT, ET, and/or surgery			- 20/52 (38.5%) showed a decrease in the sum of their intracranial lesions
						- 10/52 (19.2%) showed stable disease for ≥6 months
						- Intracranial benefit rate of 25%
	MONARCH-1 [1]	Phase II	- HR+/HER2-	Abemaciclib alone	- Median PFS = 6.0 months	- *Excluded patients with CNS metastases*
			- Metastatic		- Median overall survival = 17.7 months	
			- Progressed on ET and CT			
	MONARCH-2 [1]	Phase III	- HR+/HER2-	Fulvestrant+abemaciclib vs. fulvestrant+placebo	- PFS benefit = 7.1 months (16.4 vs. 9.3 months)	- *Excluded patients with CNS metastases*
			- Metastatic			
			- Progressed on ET			
	MONARCH-3 [1]	Phase III	- HR+/HER2-	Non-steroidal AI+abemaciclib vs. non-steroidal AI+placebo	- ORR = 59% vs. 44%	- *Excluded patients with CNS metastases*
			- Advanced/Metastatic		- PFS benefit >10.8 months; not reached (>25.5 months) vs. 14.7 months	
			- Treatment-naïve			
Ribociclib	MONALEESA-2 [1]	Phase III	- HR+/HER2-	Letrozole+ribociclib vs. letrozole+placebo	- 18 month PFS rate = 63.0% vs. 42.2%	- *Excluded patients with CNS metastases*
			- Advanced/Metastatic		- ORR = 52.7% vs. 37.1%	
			- Treatment-naïve		- PFS benefit >8.3 months; not reached (>23 months) vs. 14.7 months	
	MONALEESA-7 [1]	Phase III	- HR+/HER2-	Tamoxifen or non-steroidal AI+goserelin+ribociclib vs. tamoxifen or non-steroidal AI+goserelin+placebo	- PFS benefit = 10.8 months (23.8 vs. 13.0 months)	- *Excluded patients with CNS metastases*
			- Advanced/Metastatic			
			- Progressed on ET and/or CT			
	MONALEESA-3 [11]	Phase III	- HR+/HER2-	Fulvestrant+ribociclib vs. fulvestrant+placebo	- PFS benefit = 7.7 months (20.5 vs. 12.8 months)	- 8 patients with known brain metastases (stable, previously treated)
			- Advanced/Metastatic		- ORR = 40.9% vs. 28.7%	- 6/8 patients in the ribociclib arm
			- Treatment-naïve or progressed on ET			- 2/8 patients in the placebo arm
						- No specific outcome data available for these patients
Palbociclib	PALOMA-1 [1]	Phase II	- HR+/HER2-	Letrozole+palbociclib vs. letrozole alone	- PFS benefit = 10 months (20.2 vs. 10.2 months)	- *Excluded patients with CNS metastases*
			- Advanced/Metastatic			
			- Treatment-naïve			
	PALOMA-2 [9]	Phase III	- HR+/HER2-	Letrozole+palbociclib vs. letrozole+placebo	- PFS benefit = 10.3 months (24.8 vs. 14.5 months)	- 2 patients with known brain metastases (stable after RT)
			- Advanced/Metastatic			- 9 patients developed new brain lesions while on study protocol
			- Treatment-naïve			- *Details could not be disclosed due to concerns regarding patient de-identification*
	PALOMA-3 [10]	Phase III	- HR+/HER2-	Fulvestrant+palbociclib vs. fulvestrant+placebo	- PFS benefit = 5.4 months (9.2 vs. 3.8 months)	- 5 patients with known brain metastases (stable, no prior RT)
			- Advanced/Metastatic			- 2 patients developed new brain metastases while on study protocol
			- Progressed on ET			- *Details could not be disclosed due to concerns regarding patient de-identification*

AI: aromatase inhibitor; ET: endocrine therapy; CT: chemotherapy; RT: radiotherapy; PFS: progression free survival; ORR: objective response rate.

Among trials evaluating the efficacy of CDK4/6 inhibitors in patients with treatment-naïve HR+/HER2- advanced breast cancer, only PALOMA-2 included patients with brain metastases (2 of 666 enrolled participants), but only if they were previously treated and stable. In this trial, one patient was randomized to each treatment arm and neither had progression of brain metastases during a median follow-up of 23 months. However, 5 patients (1.1%) in the palbociclib arm and 4 (1.8%) patients in the placebo arm developed new brain lesions during the trial [9]. Other phase III studies, MONALEESA-2 and MONARCH-3, and the phase II PALOMA-1 study demonstrated a PFS benefit for CDK4/6 inhibitors in HR+/HER2- advanced breast cancer (Table 1), but they all excluded patients with brain metastases; unfortunately, sites of disease progression were not available in these studies [1].

For patients with HR+/HER2- advanced breast cancer whose disease progressed on one or more lines of treatment, several studies demonstrated a PFS benefit for CDK4/6 inhibitors plus endocrine therapy (Table 1). However, only PALOMA-3 included patients with brain metastases [10], with a requirement that brain metastases had to be treated and stable. A total of 5 patients with brain metastases were included in the study, and 2 other patients developed new brain metastases while on the study protocol (Table 1). Unfortunately, details regarding the outcomes of these patients could not be disclosed due to concerns regarding patient de-identification. Other comparable phase III trials, MONALEESA-7 and MONARCH-2, excluded patients with CNS metastases and sites of disease progression were not recorded [1]. However, MONALEESA-3, a phase III study for patients with HR+/HER2- advanced breast cancer treated with ribociclib in the first or second-line setting included patients with treated and stable brain metastases. A total of 8 patients with brain metastases were included, 6 in the ribociclib arm and 2 in the placebo arm [11], but CNS specific outcomes were not available.

### Evidence from pre-clinical models

CDK4/6 inhibitors, first investigated for treatment of glioblastoma multiforme (GBM), were shown to cross the BBB in mouse and rat models [3, 6]. Interestingly, radioactively labeled abemaciclib demonstrated that radioactivity from ^14^C could be detected in CNS tissues up to 12 hours after a single oral dose of 10 mg/kg [3]. A Phase I trial further demonstrated that concentrations of abemaciclib could be measured in the plasma and cerebrospinal fluid of patients [12]. Though CDK4/6 inhibitors have not been used to treat CNS metastases in breast cancer xenograft models, they have demonstrated efficacy for the treatment of GBM [3, 6] and brainstem glioma [4]; specifically, palbociclib in addition to radiotherapy showed greater efficacy compared to radiotherapy alone [4]. Interestingly, abemaciclib has also demonstrated anti-tumour activity in other pre-clinical models, such as non-small cell lung cancer, mantle cell lymphoma, lung cancer, acute myeloid leukemia, and colorectal cancer [5]. This raises the possibility that CDK4/6 inhibitors may have CNS activity among patients with a variety of metastatic malignancies.

## DISCUSSION

Though recent phase III trials demonstrated a PFS and even an overall survival benefit for CDK4/6 inhibitors for advanced HR+/HER2- breast cancer in the first or second-line setting [13, 14], there is limited evidence to inform their CNS-specific activity. In PALOMA-2 and PALOMA-3, patients with stable and treated brain metastases were included, but the incidence of new or progressive disease in the brain was too low to confidently assess CNS activity. Unfortunately, studies that excluded patients with brain metastases did not record the specific sites of progression, and thus, the potential utility of CDK4/6 inhibitors for the prevention of CNS metastases remains unknown. The exclusion of patients with CNS metastases in most clinical studies may be attributed to difficulty in the assessment of CNS-specific response or progression, since multiple factors including prior lines of therapy, radiotherapy, and/or exposure to corticosteroids can influence neurologic symptoms and imaging-based response. Furthermore, patients with CNS metastases represent a subset of the metastatic breast cancer population with a very poor prognosis, which may be attributed to biologically aggressive disease [15] that is capable of crossing the BBB and initiating distant CNS metastases. The increased risk of death among patients with brain metastases and the potential for CNS disease progression (potentially with systemic stability/response) may complicate the evaluation of investigational new agents in clinical trials. However, given that preliminary data from NCT02308020 and several pre-clinical studies suggest some CNS activity of CDK4/6 inhibitors, inclusion of patients with brain metastases in relevant clinical studies is warranted, ideally with appropriate assessment of CNS-specific responses, as well as measures of CNS toxicity, functional status/neurologic quality of life and patient-reported outcomes [8]. In fact, Friends of Cancer Research, a non-profit organization in the United States, recently published recommendations to broaden clinical trial eligibility across tumor subtypes to include patients with brain metastases.

Until recently, CDK4/6 inhibitors have not been investigated in patients with HER2+ or triple negative breast cancers, which portend the highest risk of brain metastases. NCT02657343 is a phase Ib/II trial for ribociclib in combination with T-DM1 or Trastuzumab in HER2+ advanced breast cancer that unfortunately excludes patients with unstable or recently treated CNS metastases. However, the NCT02978716 phase II trial of Gemcitabine/Carboplatin +/− Trilaciclib in locally recurrent or metastatic triple negative breast cancer allows the enrollment of patients with CNS metastases. The results of these new trials may help elucidate CNS-specific activity of CDK4/6 inhibitors, but conclusive data is unlikely to arise imminently given the timelines of these trials. Innovative studies that assess CNS-specific PFS and/or time-to-CNS progression after local therapy with or without the addition of promising systemic agents may help inform a future generation of clinical trials for the prevention and treatment of brain metastases.

## MATERIALS AND METHODS

To determine which studies were relevant for inclusion in our analysis, we performed a literature search of phase I, II or III clinical trials that investigated the use of CDK4/6 inhibitors for the treatment of advanced/metastatic HR+ breast cancer. Published abstracts were also included. For published trials that did not include information on CNS metastases, we submitted requests for unpublished data to the company sponsors. We also performed a search on the clinicaltrials.gov database for active clinical trials that met our search criteria.

## Abbreviations

AI: aromatase inhibitor
BBB: blood-brain barrier
CDK4/6: cyclin dependent kinase 4/6
CNS: central nervous system
CT: chemotherapy
ET: endocrine therapy
GBM: glioblastoma multiforme
HR: hormone receptor
ORR: objective response rate
PFS: progression free survival
RT: radiotherapy
